# Measurement of expectations regarding exercise therapy of patients with hip and knee osteoarthritis: A scoping review

**DOI:** 10.1016/j.ocarto.2025.100617

**Published:** 2025-04-25

**Authors:** A.P.M. Konings-Pijnappels, M.C. van der Steen, H. Seetsen-van Schelven, I. Hoogendoorn, T.P.M. Vliet Vlieland, R.P.A. Janssen

**Affiliations:** a Department of Health Innovations and Technology, Fontys University of Applied Sciences, Eindhoven, the Netherlands; b Department of Orthopaedic Surgery & Trauma, Máxima Medical Centre, Eindhoven-Veldhoven, the Netherlands; c Department of Orthopaedic Surgery & Trauma, Catharina Hospital, Eindhoven, the Netherlands; d Department Research and Medical Innovation, Máxima Medical Centre, Eindhoven-Veldhoven, the Netherlands; e Leiden University Medical Center (LUMC), Leiden, the Netherlands; f Department of Biomedical Engineering, Eindhoven University of Technology, Eindhoven, the Netherlands

**Keywords:** Hip osteoarthritis, Knee osteoarthritis, Expectations, Exercise therapy

## Abstract

**Objective:**

While exercise therapy generally yields positive outcomes in patients with hip or knee osteoarthritis (HOA or KOA), individual results vary. Expectations may influence treatment results, but research on this topic is scarce. Therefore, this study aimed to describe methods to assess pre-treatment expectations of patients with HOA or KOA with respect to exercise therapy.

**Design:**

Scoping review, with the Pubmed, Embase, CINAHL and Cochrane databases searched for original clinical studies of any design reporting a method or instrument to assess expectations of exercise therapy in patients with HOA or KOA. Data extraction from selected studies concerned study characteristics and the method or instrument used, as well as their content, categorized into expectations of structure, process or outcomes of exercise therapy treatment.

**Results:**

Twenty-nine studies met the inclusion criteria. Twenty-three different methods used to address expectations were identified, 3 of which had a qualitative nature (interviews) and 20 concerned questionnaires. There was a large variation in number of items and measurement scales in the identified methods. Nine methods addressed expectations of outcomes of treatment, 7 addressed both outcomes and the process/structure of treatment. One instrument was found to measure willingness to receive exercise rather than expectations. For the remaining 6 instruments no content nor aspects measured were reported.

**Conclusion:**

In the literature, apart from interviews, mainly quantitative methods have been used to measure expectations with regard to exercise therapy in patients with HOA or KOA. Most of their content concerned the outcomes of care, rather than its structure and/or process.

## 1 Introduction

Osteoarthritis of the hip and knee (HOA and KOA) are two common and disabling conditions for which there is currently no cure. Total hip and knee surgery are effective treatment options in end stage HOA and KOA [1]. In earlier stages, there are various pharmacological and non-pharmacological treatment options available [2]. Exercise and exercise therapy are important treatment modalities, which seem to result in reduction of pain and improvement of physical function and quality of life [[3], [4], [5]]. Studies report various exercise therapies that differ in terms of duration, supervision, and content [[3], [4], [5]]. Supervision by physical therapists is recommended regarding pain management and treatment performance [6,7]. Despite overall favourable results, the treatment response varies among individual patients [4,8].

Several determinants of the effect of exercise in individual patients with HOA or KOA have been identified, including Body Mass Index, age, gender, comorbidities and number of face to face contact occasions with a caregiver [4,[8], [9], [10]]. So far, the role of treatment expectations for osteoarthritis, as a determinant of outcomes of exercise therapy, has not been explored. This is striking as particular in surgical treatment of HOA and KOA, the role of expectations has been a topic of interest. Studies showed, however, contradictory results [[11], [12], [13]]. One study concluded that there is no correlation between pre-operative expectations and post-operative Patient Reported Outcome Measures such as satisfaction, symptoms and disability and quality of life [12]. Whereas others report higher expectations to be associated with better function and less pain after surgery [11] and expectations that are too high lead to more disappointment after treatment [13]. Studies regarding expectation of exercise therapy for lower back pain showed the influence of healthcare providers and previous patient experience on perception of treatment [14,15].

Expectations are generally considered to be about one's own treatment, whereas beliefs or perceptions are conceptualized as expectations that people have in general. These terms are sometimes used interchangeably [14,16]. Moreover, expectations form a multi-dimensional concept [17] which can be related to aspects of behaviour and quality of care. Several models or theories can be used to categorize these aspects, such as biopsychosocial model [18], health belief model [19], theory of planned behaviour [20] and approaches for assessment of quality of care [21]. Utilization of the, frequently narrowly conceptualized, biopsychosocial model might induce a limited description of expectations [18]. Unlike the health belief model and the theory of planned behavior, which focus on explaining or predicting behavior, this review emphasizes expectations concerning multiple aspects of care. Therefore utilization of the aspects of quality of care as provided by Donabedian (1988) is better suitable [21]. These aspects are defined; structure (the system), process (actual activities) and outcome (effect) of care [21].

Given the lack of knowledge on the role of expectations on exercise therapy in HOA and KOA the current review aimed to explore how pre-treatment expectations of exercise therapy have been assessed in the literature and to describe methods assessing pre-treatment expectations of patients with HOA or KOA with respect to exercise therapy. More insight into the assessment methods for pre-treatment expectations of exercise therapy for HOA and KOA is relevant for both clinicians and researchers in the field of exercise therapy for HOA or KOA. Knowledge about the characteristics of the instruments, helps clinicians to decide which measure to utilize to assess expectations. In addition, researchers aiming to determine the impact of expectations on treatment outcomes may use the information to determine how variation regarding the measurement methods influences their estimation.

## 2 Methods

### 2.1 Study design

This scoping review was conducted in line with the PRISMA extension for scoping reviews guidelines [22]. The objectives, inclusion criteria and methods for this scoping review were specified in advance.

### 2.2 Search strategy

Search terms and constructs were selected in consultation with an information specialist from Máxima Medical Centre, Eindhoven-Veldhoven, The Netherlands. The following terms, including synonyms and closely related words, were used as index terms or free-text words: expectations, conservative treatment, knee or hip osteoarthritis. The full search strategy is available in appendix 1. On November 24th, 2022, the information specialist performed a systematic search in literature databases of Pubmed, Embase, CINAHL and Cochrane. An update was performed on April 10th, 2024. The yield of the search was entered into Endnote, whereas duplicates were removed using the Bramer method [23]. All unique records were further processed in Rayyan (rayyan.qrci.org), a research platform for collaboration in reviews [24].

### 2.3 Eligibility criteria

The inclusion and exclusion criteria for inclusion of studies in the review are displayed in Table 1. The scoping review was confined to original clinical studies (of any design, including qualitative studies). Studies needed to include patients with HOA or KOA, who received or were about to receive any form of exercise therapy (defined as any exercises that were all or partly supervised by a physical therapist). Comparison groups, other interventions and multi-component interventions were allowed to be present In addition, expectations needed to be assessed before treatment in patients receiving or being about to receive exercise therapy. The measurement method, either qualitative or quantitative, had to be described in the study. Instruments or methods were accepted when the authors reported the use for assessment of expectations. All types of manuscripts were eligible, including conference papers, study protocols and trial registrations.

**Table 1:** Selection criteria regarding a scoping review on measurement of expectations with regard to exercise therapy of patients with hip and knee osteoarthritis.

	Inclusion criteria	Exclusion criteria
Participants	Hip and/or knee osteoarthritis	
Treatment	Exercise therapy (provided by physiotherapist)	Only medication
	Multi-component interventions were allowed	Only hip or knee replacement surgery
Parameters of interest	Prior to treatment	
	Description of method used to measure expectations	
Types of evidence	All types of manuscripts including conference abstracts, study protocols and full papers	
	Quantitative and qualitative research	
	Publication available in English or Dutch	

### 2.4 Study screening and selection

Study screening and selection followed a stepwise procedure. First, two researchers independently screened titles for eligibility, using the predefined selection criteria (HSvS and AK). In a second step, the abstracts of all selected titles were screened in a similar way. Subsequently, the full-text papers of potentially eligible papers were retrieved. In the third step, two researchers independently screened the complete texts of the included records for inclusion criteria (HSvS and AK). Any disagreement between the reviewers during the different screening phases was resolved through discussion and if necessary, a third reviewer (MvdS) was consulted. Both researchers individually registered reasons for exclusion. The main reason registered by the first author (AK) was listed in case of multiple reasons. In case multiple manuscripts about the same study were identified only the full text article was included. For example, only the full text original article was include when the author manuscript was identified as well. The study protocol was included when no original article was published or when both protocol and abstract were retrieved. The references of all included publications were screened for additional eligible studies.

### 2.5 Data extraction

A data extracting table was constructed by a researcher (AK), senior research coordinator (MvdS), orthopaedic surgeon (RJ), and professor in rehabilitation processes and physical therapy (TVV), prior to data extraction. Relevant categories were adjusted/inserted after pilot testing of the first version of the data charting table. Data extraction included the first author, country and year of publication, the localization of OA (HOA/KOA or both), age and sex distribution of the population, method to assess expectations were described by study design, how often they were identified in the search, set up and addressed aspects of care, measurement instrument (name, brief description) and its content and the timing of its administration. The term method was used to report qualitative methods such as interview guides. The term instrument was used to report quantitative methods such as questionnaires. Corresponding authors were contacted to obtain further clarification when the content of a method/instrument was ambiguous (background information did not resolve this ambiguity) or when it was unclear whether the instrument was utilized to assess expectations. One researcher performed data extraction (AK). A second researcher randomly checked data charting (MvdS) to minimise missing relevant information.

### 2.6 Data analysis

Methods and instruments used to assess expectations were categorized according to their study design, how often they were identified in the search, their set up, construct and components measured as reported and addressed aspects of care. Construct and components are reported using the terminology from the included report. The decision if an aspect was about outcome, structure and process or other aspects was based on discussion between two researchers (HSvS and AK) using the distinction reported by Donabedian [21] and Grimmer et al. [25]. A method (qualitative) or instrument (quantitative) was designated to be related to outcome if it determined the effect of treatment on e.g. pain or physical function. It was linked to structure if it addressed the system e.g. elements related to environment and relationships [21,25]. The link to process was made if the method or instrument comprised actual activities and characteristics of the treatment itself such as the competences of the physiotherapist or training facilities [21,25].

## 3 Results

### 3.1 Selection

**Fig. 1:**
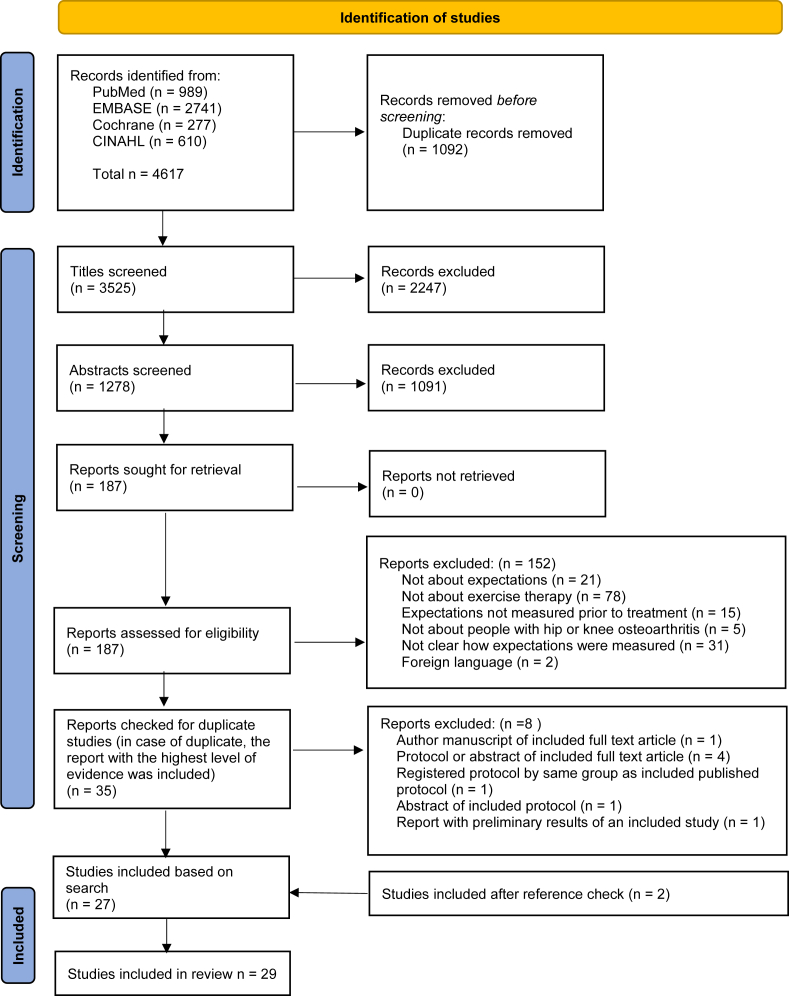
PRISMA flow chart of inclusion. n, number.

The results of the selection process are presented in Fig. 1. After screening of title and abstract from a total of 3525 records, 187 full-text reports were retrieved for further screening. Twenty-seven reports were included in this review. In addition, 2 studies were added after checking the references of the included studies. Therefore, this review comprises 29 studies (Fig. 1). Fourteen corresponding authors were contacted for further information of whom 4 responded. These studies all reported multiple instruments. The extra information of these 4 authors led to exclusion of 5 of these instruments since the authors stated that the instrument in question was not used to assess expectations. Of the other studies, it remained unclear which specific instrument was used to measure expectations. As a result these instruments were included in the current review.

### 3.2 Study characteristics

Table 2 shows all study characteristics divided in 4 categories. There were 3 qualitative original articles, 18 quantitative original articles, 3 quantitative protocols and 5 quantitative abstracts. The vast majority of the studies (22/29) focused on KOA [[26], [27], [28], [29], [30], [31], [32], [33], [34], [35], [36], [37], [38], [39], [40], [41], [42], [43], [44], [45], [46], [47]]. There were 7 studies addressing both HOA and KOA [[48], [49], [50], [51], [52], [53], [54]]. In addition, Table 2 shows that the populations in the included studies represented a broad variety of participants with regard to age, severity and duration of complaints. The populations were overall representative for general HOA or KOA populations [55]. The included studies were performed in primary care facilities, general and tertiary hospitals and non-medical facilities.

**Table 2:** Characteristics of included studies.

Author and publication year	Joint	Sample size		Age (in years)		BMI (kg/m2)				Severity of disease		Duration of complaints (in years)		Setting (type, country)
***Qualitative original article***														
Christiansen 2020 [26]	Knee	22		71 (51–96)		^‡^				NPRS 5 ± 2		55 %	≤5	Non-medical facility, USA
												45 %	^‡^	
Sharpe 2022 [27]	Knee^a^	22	Not initiated physiotherapy	60 ± 11		^‡^				^‡^		^‡^		^‡^, USA
		13	Initiated physiotherapy	58 ± 13										
		9	Not aware of physiotherapy appointment	50 ± 12										
Wallis 2020 [48]	Knee	20		70 ± 11		^‡^				^‡^		20 %	<5	^‡^, Australia
	Hip											20 %	5–10	
												20 %	11–15	
												15 %	>15	
												25 %	^‡^	
***Quantitative original article***														
Bareyre 2019 [28]	Knee	168					66 ± 6			29 ± 5	KL 0	10 [6,15]		Non-medical facility, France
										8 %	KL 1			
										9 %	KL 2			
										52 %	KL 3			
										21 %	KL 4			
Damush 2005 [29]	Knee	191		82 %50–75			^‡^			^‡^		^‡^		Non-medical facility,^‡^
				18 %≥75										
Foster 2010 [30]	Knee	70	With treatment preference	65 ± 9			23 %	Underweight or normal		9 ± 4	WOMAC pain	33 %	<1	Primary care facility, UK
							47 %	Overweight		31 ±14	WOMAC function	36 %	1–5	
							30 %	Obese		6 ± 2	Pain severity	11 %	5–10	
												20 %	≥10	
		282	Without treatment preference	63 ± 9			56 %	Underweight or normal		9 ± 4	WOMAC pain	45 %	<1	
							45 %	Overweight		30 ± 13	WOMAC function	31 %	1–5	
							35 %	Obese		6 ± 2	Pain severity	11 %	5–10	
												13 %	≥10	
Gay 2020 [31]	Knee	69	Intervention group	67 ± 6			29 ± 4			KL 3 [^‡^ ]		^‡^		Non-medical facility, France
		54	Control group	65 ± 7			29 ± 6			KL 3 [^‡^ ]		^‡^		
Hinman 2020 [32]	Knee	88	Existing service	63 ± 8			31 ± 8			^‡^		9 ± 8		^‡^, Australia
		87	Exercise advice	62 ± 9			31 ± 7					10 ± 9		
Jessep 2009 [33]	Knee	35	Outpatient physiotherapy group	67 (51–76)			29 (20–47)			^‡^		12 (1–55)		Primary care facilities, UK
		29	Rehabilitation program group	66 (53–81)			30 (20–42)			^‡^		13 (1–30)		
Lawford 2017 [49]	Knee	330		62 ± 8			^‡^			5 ± 2 pain level NRS averaged over past week		^‡^		^‡^, Australia
	Hip													
Lawford 2018 [34]	Knee	74	Intervention group	61 ± 7			32 ± 14			^‡^		^‡^		^‡^, Australia
		74	Control group	62 ± 8			30 ± 10							
Lee 2018 [35]	Knee	182		60 ± 11			32 ± 7			7 %	KL 0-1	9 ± 10		Tertiary hospital, USA
										40 %	KL 2			
										31 %	KL 3			
										22 %	KL 4			
Marszalek 2017 [36]	Knee	262		60 ± ^‡^			32 ± ^‡^			^‡^		^‡^		^‡^, USA
O'Leary 2020 [37]	Knee	114	Poor response group	63 ± 9			35 ± 8			^‡^		^‡^		Tertiary hospitals, Australia
		124	Positive response group	62 ± 12			34 ± 8							
Piyakhackornrot 2011 [38]	Knee	31	Facility based exercise program	(38–59)			24 ± 3			^‡^		32 %	<1	General hospital, Thailand
												42 %	1–5	
												26 %	>5	
		34	Supervised home-based exercise program	(38–59)			25 ± 3					17 %	<1	
												74 %	1–5	
												9 %	>5	
Quicke 2017 [39]	Knee	514		10 %	45–49		20 %		Underweight or normal	^‡^		25 %	<1	Primary care facilities, UK
				30 %	50–59		42 %		Overweight			39 %	1–5	
				36 %	60–69		39 %		Obese			19 %	5–10	
				19 %	70–79							18 %	>10	
				5 %≥	80									
Ribero 2024 [50]	Knee Hip	51	Usual care	66 ± 10			30 ± 6			86 ± 52	WOMAC	^‡^		^‡^, New Zealand
										17 ± 11	WOMAC pain			
										60 ± 39	WOMAC function			
		54	Usual care plus manual therapy	67 ± 11			29 ± 6			104 ± 56	WOMAC			
										22 ± 12	WOMAC pain			
										72 ± 42	WOMAC function			
		51	Usual care plus exercise therapy	66 ± 8			29 ± 6			83 ± 56	WOMAC			
										17 ±11	WOMAC pain			
										59 ± 41	WOMAC function			
		50	Usual care plus combined manual and exercise	65 ± 9			31 ± 6			60 ±50	WOMAC			
										20 ± 10	WOMAC pain			
										66 ± 37	WOMAC function			
Selten 2017 [51]	Knee Hip	351		63 ± 12			31 %		Normal	^‡^		11 ± 10		Tertiary hospital, Netherlands
							43 %		Overweight					
							26 %		Obese					
Selten 2018 [52]	Knee Hip	289		63 ± 11			24 %		Normal	47 ± 21	WOMAC total score	4 %	<1	Tertiary hospital, Netherlands
							49 %		Overweight	10 ± 5	WOMAC pain	37 %	1–5	
							27 %		Obese	32 ± 16	WOMAC functioning	27 %	5–10	
										5 ± 2	WOMAC stiffness	32 %	>10	
Vina 2019 [53]	Knee Hip	130	Hispanic	62 ± 8			^‡^			59 ± 20	WOMAC pain	^‡^		Tertiary hospital, USA
										61 ± 22	WOMAC stiffness			
										59 ± 21	WOMAC disability			
		232	Non-hispanic	65 ± 8						44 ± 20	WOMAC pain			
										53 ± 23	WOMAC stiffness			
										43 ± 20	WOMAC disability			
Zhou 2018 [40]	Knee	575	Non-patients	40 ± 19			^‡^			^‡^		^‡^		^‡^, China
		299	Patients	49 ± 13										
***Quantitative study protocol***														
Jakiela 2023 [41]	Knee	100		^‡^			^‡^			^‡^		^‡^		^‡^, USA
Schlenk 2011 [42]	Knee	182		65 ± 8			34 ± 7			^‡^		^‡^		^‡^, USA
Torstensen 2018 [43]	Knee	200		45–85			^‡^			KL 1-3		^‡^		Primary care facilities and tertiary hospitals, Sweden
***Quantitative abstract***														
Chang 2014 [44]	Knee	282		60 ± 10			^‡^			51 %	KL 0-2	^‡^		^‡^, USA
										22 %	KL 3			
										27 %	KL 4			
Gamache 2014 [45]	Knee	282		60 ± 10			32 ± ^‡^			^‡^		^‡^		^‡^, USA
Hasan 2018 [46]	Knee	72		^‡^			^‡^			^‡^		^‡^		General hospitals, Malaysia
Toomey 2022 [54]	Knee	98	55 %	50–69			^‡^			^‡^		^‡^		^‡^, Ireland
	Hip		45 %	^‡^										
Zhou 2018 [47]	Knee	34	^‡^				33 ± ^‡^			5 ± 2 patients global assessment score		11 ± ^‡^		^‡^, USA

Mean ± standard deviation; median [interquartile range], mean (range), ^‡^ no information available in included study, yrs years, NPRS Numeric Pain Rating Scale, KL Kellgren and Lawrence grade, WOMAC Western Ontario and McMaster Universities osteoarthritis index, NRS Numeric Rating Scale.

a This study included participants with low back pain as well as KOA.

### 3.3 Methods and instruments used to assess expectations

In total, 23 different methods or instruments used to address expectations were identified (Table 3). Three methods had a qualitative nature by means of an interview guide [26,27,48]. The other 20 were quantitative methods with questionnaires [[28], [29], [30], [31], [32], [33], [34], [35], [36], [37], [38], [39], [40], [41], [42], [43], [44], [45], [46], [47],[49], [50], [51], [52], [53], [54]]. The Outcome Expectations for Exercise questionnaire (OES) was used 7 times [35,36,39,[43], [44], [45],^47^]. Furthermore, there were 11 study-specific, custom-developed, not elsewhere reported or validated, question(naire)s [29,30,32,34,37,40,41,47,49,53,54]. There was a large variation in number of items and measurement scales in the identified methods and instruments (Table 3). Most instruments used a Likert scale. Only 3 studies reported information about validity of the questionnaire (Table 4). In these studies, the OES, Treatment Beliefs of Osteoarthritis questionnaire (TOA) and Arthritis Self-Efficacy Scale (ASES)-F showed high internal consistency [28,39,51,52]. For the TOA, high face validity [51] and high test-retest reliability [51,52] were additionally reported.

**Table 3:** Reported description of methods and instruments used to assess expectations.

Study design	Number of included studies using instrument	Method/instrument	Number of items		Measurement scale	Construct measured (as reported)	Components covered by method/instrument (as reported)	Aspect of care
*Qualitative*								
	1	Interview guide [26]	5			Barriers Facilitators	Past experience Treatment Delivery of information	Outcome/Structure/Process
		Interview guide [27]	a			(Factors influencing) choices	Referral Effectiveness Delivery of information Finance Treatment modality Past experience Type of medical provider	Outcome/Structure/Process
		Interview guide [48]	a			Perceptions	Understanding of KOA and HOA and management including GLA:D Current and previous management Views on participation in exercise Barriers and enablers for starting GLA:D Barriers and enablers for continuing GLA:D	a
*Quantitative*								
	7	Outcome expectations for exercise questionnaire (OES) [35,36,39,[43], [44], [45],^47^]	9		5-Point likert	Expectations [39,40,44,45] Perceptions [35] Beliefs [35,36,43] Attitude [43])	a	Outcome
	3	Treatment beliefs of osteoarthritis (TOA) [41,51,52]	60		5-Point likert	Beliefs [41,52]	Physical activity [51,52]; Physiotherapy [51,52]; Pain medication [51,52]; Injections [51,52]; Arthroplasty [51,52]	Outcome [51,52]
	2	Exercise health beliefs and self-efficacy (ExBeliefs) [33,46]	20		5-Point likert	Beliefs [33] Self-efficacy [33]	a	a
		Knee osteoarthritis fears and beliefs questionnaire (KOFBeQ) [28,31]	a		a	Beliefs [31] Fears [31] Self-efficacy [28]	a	a
		Self-efficacy for exercise scale (SEE) [38,43]	15		No, uncertain, yes	Beliefs [43] Attitude [38,43]	a	a
	1	Arthritis self efficacy scales for pain, function and other symptoms- French version (ASES-F) [28]	20		1-10 NRS	Self-efficacy	Pain self-efficacy Function self-efficacy Other symptoms self-efficacy	Outcome
		Evaluation of the perception of physical activity (EPPA) [31]	a		a	a	a	a
		Pain belief screening instrument [50]	7		0-10 scale	Beliefs	Pain intensity Disability Fear Avoidance Catastrophizing thinking Self-efficacy	Outcome
		Perceived therapeutic efficacy scale for arthritis (PTES (-a)) [42]	a		a	Expectations^b^	a	a
		Study specific, custom developed questionnaire [37]	1		VAS	Expectations	a	Outcome
		Study specific, custom developed questionnaire [41]	1		a	Beliefs Self-efficacy	Treatment expectations	Outcome
		Study specific, custom developed questionnaire [40]	1		4-Point likert	Perceptions	a	Other aspects
		Study specific, custom developed questionnaire [32]	1		5-Point likert	Expectations	Improvement	Outcome
		Study specific, custom developed questionnaire [34]	1		5-Point likert	Expectations	a	Outcome
		Study specific, custom developed questionnaire [30]	5		11-Point NRS and 4-point likert	Expectations	Improvement of pain, movement and function	Outcome
		Study specific, custom developed questionnaire [54]	6		5-Point likert	Beliefs	Improvement Safety Effectiveness	Outcome/Structure/Process
		Study specific, custom developed questionnaire [53]	8		Yes/no 5-Point likert	Beliefs Attitude	Understanding of exercise benefit and risk of exercise	Outcome/Structure/Process
		Study specific, custom developed questionnaire [49]	17		5-Point likert	Perceptions	Treatment modality Safety Effectiveness Acceptability Affordability/finances	Outcome/Structure/Process
		Study specific, custom developed questionnaire [29]	20		4-Point likert	Motivation	Treatment modality Relevant others Finance Improvement/effectiveness reimbursement/gifts Self-efficacy	Outcome/Structure/Process
		Study specific, custom developed questionnaire [40]	24		6-Point likert	Perceptions	Effectiveness (improvement of) pain Safety Recommendation Responsibility Decision making Treatment modality Education	Outcome/Structure/Process

NRS numeric rating scale; VAS visual analogue scale range 1–10.

a No information available in included study.

b Based on additional communication with author.

**Table 4:** Reported psychometric properties.

	Internal consistency		Face validity	Test-retest reliability (ICC)
	Cronbach's alpha	Pearson correlation coefficient		
Outcome expectations for exercise questionnaire (OES) [39]	0.80–0.93	0.61–0.69	b	b
Treatment beliefs of osteoarthritis (TOA) [51,52]	0.66–0.90	b	High	0.66–0.88
Arthritis self-efficacy scale (ASES)-F [28]	0.95	b	b	0.84^a^

a Lin concordance coefficient.

b No information available in included study.

### 3.4 Measured constructs as reported

The main focus of this review was on the construct expectations/perceptions and beliefs, which were explicitly reported to be measured in 18 methods and instruments [28,[30], [31], [32], [33], [34], [35], [36], [37],[39], [40], [41], [42], [43], [44], [45],[47], [48], [49], [50], [51], [52], [53], [54]]. A further 2 methods and instruments were reported to measure constructs related to expectations; namely Barriers and facilitators and (factors influencing) choices [26,27]. There were also 8 instruments that were related to other constructs such as attitudes, fears, self-efficacy and motivation [28,29,31,33,38,41,43,53]. Of these, 6 instruments were reported to cover constructs related to expectations as well [28,31,33,35,36,38,39,41,[43], [44], [45], [46], [47],^53^]. The remaining 2 instruments were reported to only focus on self-efficacy [28] or motivation [29] (Table 3), even though the articles in which these instruments were used reported to address expectations.

### 3.5 Components covered as reported

The included studies reported a variety of components covered by their method or instrument (Table 3). Fourteen methods and instruments reported components related to effectiveness (outcome) and modality (structure/process) of exercise therapy [[26], [27], [28], [29], [30],^32^,^40^,^41^,[48], [49], [50], [51], [52], [53], [54]]. Of these 14 instruments, 3 covered also components of additional treatments such as pain medication, injections, arthroplasty [51,52] and components of self-efficacy [29,50] alongside components on outcome and structure/process. Only the study of Bareyre et al. covered merely self-efficacy [28]. For the remaining 9 instruments, no content was reported in the included studies [28,31,[33], [34], [35], [36], [37], [38], [39], [40],[42], [43], [44], [45], [46], [47]].

### 3.6 Aspects of care

Nine instruments only addressed expectations with regard to the aspect outcome of treatment such as pain, physical activity or use of medication [28,30,32,[34], [35], [36], [37],^39^,^41^,[43], [44], [45],^47^,[50], [51], [52]]. Seven other methods and instruments addressed both aspects of outcome of treatment and process/structure of treatment [26,27,29,47,49,53,54]. One instrument was about a different aspect, willingness to receive therapy [40]. Six methods and instruments did not report the aspects measured with the instrument [28,31,33,38,42,43,46,48] (Table 3).

## 4 Discussion

This scoping review aimed to identify and describe the methods to assess expectations of patients with HOA and KOA before starting exercise therapy. Twenty-nine studies were selected, in which 23 methods or instruments used to assess expectations of exercise therapy were identified. Three of these had a qualitative nature, i.e. used an interview guide. For the remaining 20 quantitative instruments, the OES and TOA were used most often. The other 18 quantitative instruments were a broad variety of instruments, which were mostly custom developed questionnaires for a specific study.

The identified methods and instruments are mainly used to measure expectations and related constructs such as beliefs, perceptions, barriers/facilitators. Expectations are generally considered to be about one's own treatment, whereas beliefs or perceptions are conceptualized as expectations that people have in general. Since these terms are used interchangeably [14,16], the current review considered them all as construct of expectations. Most of the identified methods and instruments focus on effectiveness and process of treatment. The OES was specifically developed to measure outcome expectations of exercise therapy [56], whereas TOA was developed for OA specifically but considered the broader construct of treatment beliefs in this patient group [51,52]. Both the OES and TOA were found to be reliable and valid instruments for measuring expectations [39,51,52]. However, instruments measuring different constructs and components, such as self-efficacy and use of pain medication were identified as well. One could therefore question if all identified methods and instruments -by the authors of the included studies reported to be used to assess expectations-truly measure expectations related to exercise therapy. Some of these instruments were originally developed to describe constructs such a self-efficacy, fears [56,57], or the construct of osteoarthritis itself [57]. Future research should clarify to what extent these instruments are reliable and valid to measure expectations.

Exercise therapy provided by physiotherapists and physical activity in general with regard to HOA and KOA, have occasionally been investigated together [58,59]. Physical activity, as part of lifestyle advice, is part of the first step in a widely used stepped care model in the Netherlands [2]. Exercise therapy is part of the second step in that model [2]. The focus of this review was on exercise therapy supervised by physiotherapists. As such, the current review only included studies with regard to exercise therapy provided by physiotherapists. The TOA was specifically developed for health beliefs related to physical therapy and for the promotion of physical activity [41] in physiotherapy or various other treatment modalities [51]. Therefore, it can be seen as suitable for measuring expectations in patients starting with exercise therapy, especially with its subscale on physiotherapy. Some of the other instruments were developed for exercise or physical activity in general [56,60,61]. They were however used to measure expectations in patients starting with supervised exercise therapy and therefore included in the current review. Further research is necessary to investigate the suitability of these instruments to measure expectation related to exercise therapy.

The analysis of the present review used the distinction for aspects related to outcome, process and structure of care as provided by Donabadian [21]. The vast majority of the methods and instruments focused on patients’ expectations regarding the outcomes of treatment, half of the time in combination with expectations on the structure and process of care. The observation that most of the instruments used for measuring expectations focused on aspects of outcomes, is in line with the methodology employed in studies on outcome of orthopedic treatment of patients with OA, such as hip and knee arthroplasty. Similarly, these studies used quantitative instruments with a broad variety in content. For example, the Hospital for Special Surgery knee surgery and knee replacement expectations surveys (HSS) [62] and Credibility Expectancy Questionnaire [63] focus on improvement of symptoms. The identified instruments in the current review addressed expectations related to multiple aspects of outcome such as pain, physical activity as well as physical and mental well-being. These different aspects of outcome are considered important to take into account when evaluating the effect of treatment [64]. As such, methods to assess expectations on outcome are available in the literature.

Considering the multi-dimensional concept of care and related expectations, the focus on outcome of treatment seems too narrow when measuring expectations [21,25,[65], [66], [67]]. The aspects process and structure remained underexposed in the identified methods and instruments of the present review. When structure and process of care were addressed, a variety of topics were assessed such as the relationship between patient and professional, accessibility of care, safety, time investment and enjoyability of the program [56,57,65,66]. Literature concerning the impact of process and structure aspects on factors affecting expectations and experienced quality of care is inconsistent. According to Østerås et al., factors related to process, such as the delivery of information about disease and treatment, as well as outcome such as function and pain, are important when evaluating quality of care [68]. Gustavsson et al. report differences in how treatment delivery methods relate to process, e.g. the perceived importance of patient involvement in decision-making and the extent to which patients' expectations were met [69]. Strikingly, Gustavsson et al. also report that despite variation in process, e.g the role of the care professional, there was no difference in patients’ perception of having received good quality of care [69]. Further research on the influence of these factors is needed to determine the importance of measuring them in relation to expectations.

Some limitations of the current review should be noticed. Firstly, it is possible that relevant methods or instruments to measure expectations were missed if these were part of uncompleted studies. This risk was minimized by performing an extensive search including all kinds of study designs and reports, including research protocols and conference abstracts. Secondly, included studies did not always report the content of the method or instrument and which of the reported instruments measured expectations. Information on suitability for measuring expectations could only be reported for the OES and TOA. This was not possible for the other identified methods and instruments. Although background literature about these latter instruments was consulted and authors were contacted for clarification, content and intended use of the methods and instruments could not always be determined.

In conclusion, mainly quantitative methods are used to measure expectations with regard to exercise therapy in patients with HOA and KOA. Most of their content concerned outcome of care, rather than its structure and process. Further research should investigate the importance of measuring these latter aspects. Both the OES and TOA are shown to be reliable instruments for measuring expectations in patients with HOA and KOA. These might, however, be too extensive for clinical use. Reliability, validity and suitability of the other methods and instruments need to be investigated before their use might be recommended.

## Author contributions

All authors state to have 1) substantially contributed to the work and 2) drafting or critically reviewing the work and 3) giving final approval of the version to be published and 4) agree to be accountable for all aspects of the work. This work is licensed under CC BY 4.0.

APM Konings-Pijnappels: Conception and design; Collection and assembly of data; Analysis and interpretation of data; Drafting of the article; Obtaining of funding.

MC van der Steen: Conception and design; Collection and assembly of data; Analysis and interpretation of data; Drafting of the article; Critical revision of the article; Obtaining of funding.

H Seetsen-van Schelven: Analysis and interpretation of data; Collection and assembly of data; Critical revision of the article.

I Hoogendoorn: Critical revision of the article; Performing database search; Collection and assembly of data.

TPM Vliet Vlieland: Conception and design; Critical revision of the article; Obtaining of funding.

RPA Janssen: Conception and design; Drafting of the article; Critical revision of the article; Obtaining of funding.

## Declaration of generative AI and AI-assisted technologies in the writing process

The authors used generative AI (Copilot) in the writing process of this publication to enhance the syntactic structure of English language.

## Role of the funding source

Funded by the 10.13039/501100003246Dutch Research Council (NWO); 023.018.039.

## Declaration of competing interest

The authors state to have no competing interests.
