# Supplementary material for: Measurement of expectations regarding exercise therapy of patients with hip and knee osteoarthritis: A scoping review
Source: Osteoarthr Cartil Open. 2025 Apr 25;7(3):100617. doi: 10.1016/j.ocarto.2025.100617 (PMC12143623; doi:10.1016/j.ocarto.2025.100617)
Supplement: Multimedia component 1 [file mmc1.docx]

**Appendix 1; full search strategy**

### EMBASE (using Embase.com) on April 10^th^, 2024

/exp = thesaurus term including narrower terms. *= wild card. NEAR/n = proximity search within n-words. :ti,ab,kw = search in title abstract and author keywords.

| No. | Query | Results |
| --- | --- | --- |
| #4 | #1 AND #2 AND #3 | 2.741 |
| #3 | 'expectation'/exp OR 'experience'/exp OR 'motivation'/exp OR 'attitude to health'/exp OR 'attitude to disability'/exp OR 'health behavior'/de OR 'health belief'/exp OR 'health belief model'/exp OR 'attitude to illness'/exp OR 'patient attitude'/de OR 'expectation*':ti,ab,kw OR 'experience*':ti,ab,kw OR 'motivation*':ti,ab,kw OR ((attitude* NEAR/3 health*):ti,ab,kw) OR ((health NEAR/3 knowledge*):ti,ab,kw) OR ((attitude* NEAR/3 disabilit*):ti,ab,kw) OR ((behavior*r* NEAR/3 health*):ti,ab,kw) OR ((health NEAR/3 belief*):ti,ab,kw) OR 'predication*':ti,ab,kw OR 'satisfaction*':ti,ab,kw OR 'assumptions*':ti,ab,kw OR 'beliefs':ti,ab,kw OR 'calculation*':ti,ab,kw OR 'presupposition*':ti,ab,kw OR 'presumption*':ti,ab,kw OR 'disincentive*':ti,ab,kw OR 'incentive*':ti,ab,kw OR ((attitude* NEAR/3 illness*):ti,ab,kw) OR ((attitude* NEAR/3 patient*):ti,ab,kw) OR 'sick role*':ti,ab,kw OR (patient:ti,ab,kw AND ((acceptance NEAR/3 care*):ti,ab,kw)) | 3.330.872 |
| #2 | 'conservative treatment'/de OR 'kinesiotherapy'/exp OR 'physiotherapy'/exp OR 'health program'/exp OR 'physical activity'/exp OR 'training'/exp OR 'exercise'/exp OR 'lifestyle modification'/exp OR ((((conservative NEAR/3 management*):ti,ab,kw) OR ((conservative NEAR/3 therap*):ti,ab,kw) OR ((conservative NEAR/3 treatment*):ti,ab,kw) OR ((nonoperative NEAR/3 treatment*):ti,ab,kw) OR ((nonsurgical NEAR/3 treatment*):ti,ab,kw) OR ((conservative NEAR/3 care*):ti,ab,kw) OR 'corrective exercise*':ti,ab,kw OR 'exercise movement technique*':ti,ab,kw OR ((exercise* NEAR/3 therap*):ti,ab,kw) OR ((exercise* NEAR/3 treatment*):ti,ab,kw) OR ((kinesi*therapeutic* NEAR/3 intervention*):ti,ab,kw) OR ((kinesi*therapeutic* NEAR/3 method*):ti,ab,kw) OR ((kinesi*therapeutic* NEAR/3 procedure*):ti,ab,kw) OR ((kinesi*therapeutic* NEAR/3 technique*):ti,ab,kw) OR ((kinesi*therapeutic* NEAR/3 treatment*):ti,ab,kw) OR ((kinesi*therapeutic* NEAR/3 exercise*):ti,ab,kw) OR 'kinesi*therap*':ti,ab,kw) AND ((physical NEAR/3 therap*):ti,ab,kw)) OR ((physical NEAR/3 treatment*):ti,ab,kw) OR 'physio therap*':ti,ab,kw OR 'physiotherap*':ti,ab,kw OR 'health care program*':ti,ab,kw OR 'health program*':ti,ab,kw OR 'healthcare program*':ti,ab,kw OR 'physical activit*':ti,ab,kw OR 'climbing':ti,ab,kw OR 'cycling':ti,ab,kw OR 'fighting':ti,ab,kw OR 'jogging':ti,ab,kw OR 'jumping':ti,ab,kw OR 'lifting effort':ti,ab,kw OR 'nordic walking':ti,ab,kw OR 'racewalking':ti,ab,kw OR 'running':ti,ab,kw OR 'stretching':ti,ab,kw OR 'swimming':ti,ab,kw OR 'walking':ti,ab,kw OR 'weight lifting':ti,ab,kw OR ((athletic* NEAR/3 training*):ti,ab,kw) OR 'detraining*':ti,ab,kw OR ((physical* NEAR/3 training*):ti,ab,kw) OR ((sport* NEAR/3 training*):ti,ab,kw) OR ((technical* NEAR/3 training*):ti,ab,kw) OR ((training NEAR/3 athlete*):ti,ab,kw) OR ((training NEAR/3 course*):ti,ab,kw) OR ((training NEAR/3 program*):ti,ab,kw) OR ((biometric NEAR/3 exercise*):ti,ab,kw) OR ((exercise* NEAR/3 capacit*):ti,ab,kw) OR ((exercise* NEAR/3 performance*):ti,ab,kw) OR ((exercise* NEAR/3 training*):ti,ab,kw) OR 'exertion':ti,ab,kw OR ((fitness NEAR/3 training*):ti,ab,kw) OR ((fitness NEAR/3 workout*):ti,ab,kw) OR ((physical NEAR/3 conditioning*):ti,ab,kw) OR ((physical NEAR/3 effort*):ti,ab,kw) OR ((physical NEAR/3 exercise*):ti,ab,kw) OR ((physical NEAR/3 exertion*):ti,ab,kw) OR ((physical NEAR/3 'work out*'):ti,ab,kw) OR ((physical NEAR/3 workout*):ti,ab,kw) OR 'aerobic exercise*':ti,ab,kw OR 'anaerobic exercise*':ti,ab,kw OR 'aquatic exercise*':ti,ab,kw OR 'arm exercis*':ti,ab,kw OR 'athletic performance*':ti,ab,kw OR 'breathing exercise*':ti,ab,kw OR 'calisthenics*':ti,ab,kw OR 'circuit training*':ti,ab,kw OR 'closed kinetic chain exercise*':ti,ab,kw OR 'continuous training*':ti,ab,kw OR 'cool down*':ti,ab,kw OR 'cross training*':ti,ab,kw OR 'dynamic exercise*':ti,ab,kw OR 'endurance training*':ti,ab,kw OR 'exercise intensit*':ti,ab,kw OR 'gymnastics*':ti,ab,kw OR 'intensity exercis*':ti,ab,kw OR 'interval training*':ti,ab,kw OR 'isokinetic exercise*':ti,ab,kw OR 'leg exercise*':ti,ab,kw OR 'muscle exercise*':ti,ab,kw OR 'pilates*':ti,ab,kw OR 'plyometric*':ti,ab,kw OR 'power training*':ti,ab,kw OR 'preoperative exercise*':ti,ab,kw OR 'resistance training*':ti,ab,kw OR 'squatting*':ti,ab,kw OR 'static exercise*':ti,ab,kw OR 'warm up':ti,ab,kw OR 'life style change*':ti,ab,kw OR 'life style modification*':ti,ab,kw OR 'lifestyle change*':ti,ab,kw OR 'lifestyle modification*':ti,ab,kw OR 'aquatic therap*':ti,ab,kw OR 'circuit based exercis*':ti,ab,kw OR 'dance therap*':ti,ab,kw OR 'exercise therap*':ti,ab,kw OR 'isometric exercis*':ti,ab,kw OR 'kinesiolog*':ti,ab,kw OR 'kinetic chain exercis*':ti,ab,kw OR 'motion therap*':ti,ab,kw OR 'muscle stretching exercis*':ti,ab,kw OR 'myofunctional therap*':ti,ab,kw OR 'rehabilitation exercis*':ti,ab,kw OR 'remedial exercis*':ti,ab,kw OR 'tai ji':ti,ab,kw OR 'yoga':ti,ab,kw | 1.840.409 |
| #1 | 'knee osteoarthritis'/exp OR 'knee osteoarthritis' OR 'hip osteoarthritis'/exp OR 'hip osteoarthritis' OR gonarthors*:ti,ab,kw OR ((knee NEAR/3 arthrosis*):ti,ab,kw) OR ((femorotibial NEAR/3 arthros*):ti,ab,kw) OR ((knee NEAR/3 osteoarthrit*):ti,ab,kw) OR ((knee NEAR/3 'osteo arthirit*'):ti,ab,kw) OR ((knee NEAR/3 osteoarthros*):ti,ab,kw) OR ((knee NEAR/3 'osteo arthros*'):ti,ab,kw) OR coxartheros*:ti,ab,kw OR coxarthros*:ti,ab,kw OR 'mulum coxae senilis':ti,ab,kw OR ((hip NEAR/3 arthrosis*):ti,ab,kw) OR ((cos NEAR/3 arthrosis*):ti,ab,kw) OR ((hip NEAR/3 'osteo arthrit*'):ti,ab,kw) OR ((hip NEAR/3 'osteo arthos*'):ti,ab,kw) OR ((hip NEAR/3 osteoarthros*):ti,ab,kw) OR ((hip NEAR/3 osteoarthrit*):ti,ab,kw) | 69.644 |

### PUBMED (using Pubmed.gov) on April 10^th^, 2024

[Mesh] = thesaurus term including narrower search terms. [Mesh:noExp] = thesaurus term. *= wildcard. [tiab] = search in title, abstract and author keywords.

| No. | Query | Results |
| --- | --- | --- |
| #4 | #1 AND #2 AND #3 | 989 |
| #3 | "motivation"[Mesh] OR "attitude to Health"[Mesh:noexp] OR "Health Behavior"[Mesh:NoExp] OR "Health Belief Model"[Mesh:NoExp] OR "Health Knowledge, Attitudes, Practice"[Mesh] OR "expectation*"[tiab] OR "experience*"[tiab] OR "motivation*"[tiab] OR "disincentive*"[tiab] OR "incentive*"[tiab] OR "health attitude*"[tiab] OR "attitude to health*"[tiab] OR "health knowledge*"[tiab] OR "attitude to illness*"[tiab] OR "illness attitude*"[tiab] OR "patient attitude*"[tiab] OR "sick role*"[tiab] OR "patient acceptance of care*"[tiab] OR "disability attitude*"[tiab] OR "attitude to disabilit*"[tiab] OR "health behavior*"[tiab] OR "health related behavior*"[tiab] OR "health belief*"[tiab] OR "predication*"[tiab] OR "assumptions*"[tiab] OR "beliefs"[tiab] OR "calculation*"[tiab] OR "presupposition*"[tiab] OR "presumption*"[tiab] | 2.421.477 |
| #2 | "Conservative Treatment"[Mesh] OR "Physical Therapy Modalities"[Mesh] OR "Exercise"[Mesh] OR "Exercise Therapy"[Mesh] OR "Exercise Movement Techniques"[Mesh] OR "conservative management*"[tiab] OR "conservative therap*"[tiab] OR "conservative care*"[tiab] OR "conservative treatment*"[tiab] OR "nonoperative treatment*"[tiab] OR "nonsurgical treatment*"[tiab] OR "physical therap*"[tiab] OR "physical treatment*"[tiab] OR "physio therap*"[tiab] OR "physiotherap*"[tiab] OR "exercise therap*"[tiab] OR "motion therap*"[tiab] OR "muscle stretching exercis*"[tiab] OR "aquatic therap*"[tiab] OR "kinesiology*"[tiab] OR "myofunctional therap*"[tiab] OR "biometric exercis*"[tiab] OR "exercise capacity*"[tiab] OR "exercise performanc*"[tiab] OR "exercise training*"[tiab] OR "exertion*"[tiab] OR "fitness training*"[tiab] OR "fitness workout*"[tiab] OR "physical conditioning*"[tiab] OR "physical effort*"[tiab] OR "physical exercis*"[tiab] OR "physical exertion*"[tiab] OR "physical work out*"[tiab] OR "physical workout*"[tiab] OR "aerobic exercis*"[tiab] OR "anaerobic exercis*"[tiab] OR "aquatic exercis*"[tiab] OR "arm exercis*"[tiab] OR "athletic performanc*"[tiab] OR "breathing exercis*"[tiab] OR "calisthenics"[tiab] OR "circuit training*"[tiab] OR "kinetic chain exercis*"[tiab] OR "continuous training*"[tiab] OR "cool down*"[tiab] OR "cross training*"[tiab] OR "dynamic exercis*"[tiab] OR "endurance training*"[tiab] OR "exercise intensit*"[tiab] OR "gymnastics"[tiab] OR "intensity exercis*"[tiab] OR "interval training*"[tiab] OR "isokinetic exercis*"[tiab] OR "leg exercis*"[tiab] OR "muscle exercis*"[tiab] OR "pilates"[tiab] OR "plyometric*"[tiab] OR "power training*"[tiab] OR "preoperative exercis*"[tiab] OR "resistance training*"[tiab] OR "squatting"[tiab] OR "static exercis*"[tiab] OR "warm up"[tiab] OR "physical activit*"[tiab] OR "isometric exercis*"[tiab] OR "circuit based exercis*"[tiab] OR "jogging*"[tiab] OR "swimming*"[tiab] OR "walking*"[tiab] OR "remedial exercis*"[tiab] OR "rehabilitation exercis*"[tiab] OR "exercise movement technique*"[tiab] OR "dance therap*"[tiab] OR "tai ji"[tiab] OR "yoga"[tiab] OR "athletic training*"[tiab] OR "climbing"[tiab] OR "corrective exercise*"[tiab] OR "cycling"[tiab] OR "detraining*"[tiab] OR "exercise treatment*"[tiab] OR "fighting"[tiab] OR "health care program*"[tiab] OR "health program*"[tiab] OR "healthcare program*"[tiab] OR "jumping"[tiab] OR "kinesitherapeutic exercise*"[tiab] OR "kinesitherapeutic intervention*"[tiab] OR "kinesitherapeutic method*"[tiab] OR "kinesitherapeutic procedure*"[tiab] OR "kinesitherapeutic technique*"[tiab] OR "kinesitherapeutic treatment*"[tiab] OR "kinesitherapeutical treatment*"[tiab] OR "kinesitherapy*"[tiab] OR "kinesiotherapeutic exercise*"[tiab] OR "kinesiotherapeutic intervention*"[tiab] OR "kinesiotherapeutic method*"[tiab] OR "kinesiotherapeutic procedure*"[tiab] OR "kinesiotherapeutic technique*"[tiab] OR "kinesiotherapeutic treatment*"[tiab] OR "kinesiotherapeutical treatment*"[tiab] OR "kinesiotherapy*"[tiab] OR "life style change*"[tiab] OR "life style modification*"[tiab] OR "lifestyle change*"[tiab] OR "lifestyle modification*"[tiab] OR "lifting effort"[tiab] OR "nordic walking"[tiab] OR "physical training*"[tiab] OR "racewalking"[tiab] OR "sport training*"[tiab] OR "sport specific training*"[tiab] OR "stretching"[tiab] OR "technical training*"[tiab] OR "training athlete*"[tiab] OR "training course*"[tiab] OR "training program*"[tiab] OR "weight lifting"[tiab] | 979.483 |
| #1 | "Osteoarthritis, Knee"[Mesh] OR "femorotibial arthrosis"[tiab] OR "gonarthros*"[tiab] OR "knee arthrosis"[tiab] OR "knee osteo arthrit*"[tiab] OR "knee osteo arthros*"[tiab] OR "knee osteoarthros*"[tiab] OR "knee osteoarthrit*"[tiab] OR "knee joint arthrosis"[tiab] OR "knee joint osteoarthrit*"[tiab] OR "knee joint osteo arthrit*"[tiab] OR "knee joint osteo arthros*"[tiab] OR "osteoarthritis of knee*"[tiab] OR "osteoarthritis of the knee*"[tiab] OR "Osteoarthritis, Hip"[Mesh] OR "cox arthrosis"[tiab] OR "coxartheros*s"[tiab] OR "coxarthrosis"[tiab] OR "hip arthrosis"[tiab] OR "hip osteo arthrit*"[tiab] OR "hip osteo arthros*"[tiab] OR "hip osteoarthros*"[tiab] OR "hip osteoarthrit*"[tiab] OR "hip joint osteo arthrit*"[tiab] OR "hip joint osteo arthros*"[tiab] OR "hip joint osteoarthros*"[tiab] OR "hip joint osteoarthrit*"[tiab] OR "hip joint arthrosis"[tiab] OR "malum coxae senilis"[tiab] OR "osteoarthritis of hip*"[tiab] OR "osteoarthritis of the hip*"[tiab] | 46.723 |

### Cochrane (using Wiley) on November 24^th^, 2022

MesH Descriptor: […] explode all trees = thesaurus term including narrower search terms. MesH Descriptor: […] this term only = thesaurus term. *= wildcard.

| No. | Query | Results |
| --- | --- | --- |
| #18 | #4 AND #11 AND #16 | 277 |
| #17 | #12 OR #13 OR #14 OR #15 OR #16 | 142.093 |
| #16 | expectation* OR "experience*" OR motivation* OR "disincentive*" OR "incentive*" OR "health attitude*" OR "attitude to health*" OR "health knowledge*" OR "disability attitude*" OR "attitude to disabilit*" OR "health behavior*" OR "health related behavior*" OR "health belief*" OR predication* OR "assumptions*" OR "beliefs" OR "calculation*" OR "presupposition*" OR "presumption*" | 137.991 |
| #15 | MeSH descriptor: [Health Belief Model] this term only | 41 |
| #14 | MeSH descriptor: [Health Behavior] this term only | 5.576 |
| #13 | MeSH descriptor: [Attitude to Health] this term only | 34.662 |
| #12 | MeSH descriptor: [Motivation] explode all trees | 12.262 |
| #11 | #5 OR #6 OR #7 OR #8 OR #9 OR #10 | 158.405 |
| #10 | conservative management* OR "conservative therap*" OR "conservative care*" OR "conservative treatment*" OR "nonoperative treatment*" OR "nonsurgical treatment*" OR "physical therap*" OR "physical treatment*" OR "physio therap*" OR "physiotherap*" OR "exercise therap*" OR "motion therap*" OR "muscle stretching exercis*" OR "aquatic therap*" OR "kinesiology*" OR "myofunctional therap*" OR "biometric exercis*" OR "exercise capacity*" OR "exercise performanc*" OR "exercise training*" OR "exertion*" OR "fitness training*" OR "fitness workout*" OR "physical conditioning*" OR "physical effort*" OR "physical exercis*" OR "physical exertion*" OR "physical work out*" OR "physical workout*" OR "aerobic exercis*" OR "anaerobic exercis*" OR "aquatic exercis*" OR "arm exercis*" OR "athletic performanc*" OR "breathing exercis*" OR "calisthenics" OR "circuit training*" OR "kinetic chain exercis*" OR "continuous training*" OR "cool down*" OR "cross training*" OR "dynamic exercis*" OR "endurance training*" OR "exercise intensit*" OR "gymnastics" OR "intensity exercis*" OR "interval training*" OR "isokinetic exercis*" OR "leg exercis*" OR "muscle exercis*" OR "pilates" OR "plyometric*" OR "power training*" OR "preoperative exercis*" OR "resistance training*" OR "squatting" OR "static exercis*" OR "warm up" OR "physical activit*" OR "isometric exercis*" OR "circuit based exercis*" OR "jogging*" OR "swimming*" OR "walking*" OR "remedial exercis*" OR "rehabilitation exercis*" OR "exercise movement technique*" OR "dance therap*" OR "tai ji" OR "yoga" OR "athletic training*" OR "climbing" OR "corrective exercise*" OR "cycling" OR "detraining*" OR "exercise treatment*" OR "fighting" OR "health care program*" OR "health program*" OR "healthcare program*" OR "jumping" OR "kinesitherapeutic exercise*" OR "kinesitherapeutic intervention*" OR "kinesitherapeutic method*" OR "kinesitherapeutic procedure*" OR "kinesitherapeutic technique*" OR "kinesitherapeutic treatment*" OR "kinesitherapeutical treatment*" OR "kinesitherapy*" OR "kinesiotherapeutic exercise*" OR "kinesiotherapeutic intervention*" OR "kinesiotherapeutic method*" OR "kinesiotherapeutic procedure*" OR "kinesiotherapeutic technique*" OR "kinesiotherapeutic treatment*" OR "kinesiotherapeutical treatment*" OR "kinesiotherapy*" OR "life style change*" OR "life style modification*" OR "lifestyle change*" OR "lifestyle modification*" OR "lifting effort" OR "nordic walking" OR "physical training*" OR "racewalking" OR "sport training*" OR "sport specific training*" OR "stretching" OR "technical training*" OR "training athlete*" OR "training course*" OR "training program*" OR "weight lifting" | 125.741 |
| #9 | MeSH descriptor: [Exercise Movement Techniques] explode all trees | 3.511 |
| #8 | MeSH descriptor: [Exercise Therapy] explode all trees | 21.620 |
| #7 | MeSH descriptor: [Exercise] explode all trees | 38.590 |
| #6 | MeSH descriptor: [Physical Therapy Modalities] explode all trees | 39.426 |
| #5 | MeSH descriptor: [Conservative Treatment] explode all trees | 346 |
| #4 | #1 OR #2 OR #3 | 10.699 |
| #3 | femorotibial arthrosis OR "gonarthros*" OR "knee arthrosis" OR "knee osteo arthrit*" OR "knee osteo arthros*" OR "knee osteoarthros*" OR "knee osteoarthrit*" OR "knee joint arthrosis" OR "knee joint osteoarthrit*" OR "knee joint osteo arthrit*" OR "knee joint osteo arthros*" OR "osteoarthritis of knee*" OR "osteoarthritis of the knee*" OR "cox arthrosis" OR "coxartheros*s" OR "coxarthrosis" OR "hip arthrosis" OR "hip osteo arthrit*" OR "hip osteo arthros*" OR "hip osteoarthros*" OR "hip osteoarthrit*" OR "hip joint osteo arthrit*" OR "hip joint osteo arthros*" OR "hip joint osteoarthros*" OR "hip joint osteoarthrit*" OR "hip joint arthrosis" OR "malum coxae senilis" OR "osteoarthritis of hip*" OR "osteoarthritis of the hip*" | 4.377 |
| #2 | MeSH descriptor: [Osteoarthritis, Hip] explode all trees | 1.353 |
| #1 | MeSH descriptor: [Osteoarthritis, Knee] explode all trees | 6.753 |

### CINAHL (Using EBSCO) on April 10^th^, 2024

MH "…+" = thesaurus term including narrower search terms. MH "…" = thesaurus term. *= wildcard.

| No. | Query | Results |
| --- | --- | --- |
| S4 | #s1 and #s2 and #s3 | 610 |
| S3 | MH "motivation+" OR MH "attitude to Health" OR MH "Health Behavior" OR MH "Health Belief Model" OR MH "Health Knowledge, Attitudes, Practice+" OR "expectation*" OR "experience*" OR "motivation*" OR "disincentive*" OR "incentive*" OR "health attitude*" OR "attitude to health*" OR "health knowledge*" OR "attitude to illness*" OR "illness attitude*" OR "patient attitude*" OR "sick role*" OR "patient acceptance of care*" OR "disability attitude*" OR "attitude to disabilit*" OR "health behavior*" OR "health related behavior*" OR "health belief*" OR "predication*" OR "assumptions*" OR "beliefs" OR "calculation*" OR "presupposition*" OR "presumption*" | 352.039 |
| S2 | MH "Conservative Treatment+" OR MH "Physical Therapy Modalities+" OR MH "Exercise+" OR MH "Exercise Therapy+" OR MH "Exercise Movement Techniques+" OR "conservative management*" OR "conservative therap*" OR "conservative care*" OR "conservative treatment*" OR "nonoperative treatment*" OR "nonsurgical treatment*" OR "physical therap*" OR "physical treatment*" OR "physio therap*" OR "physiotherap*" OR "exercise therap*" OR "motion therap*" OR "muscle stretching exercis*" OR "aquatic therap*" OR "kinesiology*" OR "myofunctional therap*" OR "biometric exercis*" OR "exercise capacity*" OR "exercise performanc*" OR "exercise training*" OR "exertion*" OR "fitness training*" OR "fitness workout*" OR "physical conditioning*" OR "physical effort*" OR "physical exercis*" OR "physical exertion*" OR "physical work out*" OR "physical workout*" OR "aerobic exercis*" OR "anaerobic exercis*" OR "aquatic exercis*" OR "arm exercis*" OR "athletic performanc*" OR "breathing exercis*" OR "calisthenics" OR "circuit training*" OR "kinetic chain exercis*" OR "continuous training*" OR "cool down*" OR "cross training*" OR "dynamic exercis*" OR "endurance training*" OR "exercise intensit*" OR "gymnastics" OR "intensity exercis*" OR "interval training*" OR "isokinetic exercis*" OR "leg exercis*" OR "muscle exercis*" OR "pilates" OR "plyometric*" OR "power training*" OR "preoperative exercis*" OR "resistance training*" OR "squatting" OR "static exercis*" OR "warm up" OR "physical activit*" OR "isometric exercis*" OR "circuit based exercis*" OR "jogging*" OR "swimming*" OR "walking*" OR "remedial exercis*" OR "rehabilitation exercis*" OR "exercise movement technique*" OR "dance therap*" OR "tai ji" OR "yoga" OR "athletic training*" OR "climbing" OR "corrective exercise*" OR "cycling" OR "detraining*" OR "exercise treatment*" OR "fighting" OR "health care program*" OR "health program*" OR "healthcare program*" OR "jumping" OR "kinesitherapeutic exercise*" OR "kinesitherapeutic intervention*" OR "kinesitherapeutic method*" OR "kinesitherapeutic procedure*" OR "kinesitherapeutic technique*" OR "kinesitherapeutic treatment*" OR "kinesitherapeutical treatment*" OR "kinesitherapy*" OR "kinesiotherapeutic exercise*" OR "kinesiotherapeutic intervention*" OR "kinesiotherapeutic method*" OR "kinesiotherapeutic procedure*" OR "kinesiotherapeutic technique*" OR "kinesiotherapeutic treatment*" OR "kinesiotherapeutical treatment*" OR "kinesiotherapy*" OR "life style change*" OR "life style modification*" OR "lifestyle change*" OR "lifestyle modification*" OR "lifting effort" OR "nordic walking" OR "physical training*" OR "racewalking" OR "sport training*" OR "sport specific training*" OR "stretching" OR "technical training*" OR "training athlete*" OR "training course*" OR "training program*" OR "weight lifting" | 519.016 |
| S1 | MH "Osteoarthritis, Knee+" OR "femorotibial arthrosis" OR "gonarthros*" OR "knee arthrosis" OR "knee osteo arthrit*" OR "knee osteo arthros*" OR "knee osteoarthros*" OR "knee osteoarthrit*" OR "knee joint arthrosis" OR "knee joint osteoarthrit*" OR "knee joint osteo arthrit*" OR "knee joint osteo arthros*" OR "osteoarthritis of knee*" OR "osteoarthritis of the knee*" OR MH "Osteoarthritis, Hip+" OR "cox arthrosis" OR "coxartheros*s" OR "coxarthrosis" OR "hip arthrosis" OR "hip osteo arthrit*" OR "hip osteo arthros*" OR "hip osteoarthros*" OR "hip osteoarthrit*" OR "hip joint osteo arthrit*" OR "hip joint osteo arthros*" OR "hip joint osteoarthros*" OR "hip joint osteoarthrit*" OR "hip joint arthrosis" OR "malum coxae senilis" OR "osteoarthritis of hip*" OR "osteoarthritis of the hip*" | 23.137 |
